# Foramen Ovale Closure Is a Process of Endothelial-to-Mesenchymal Transition Leading to Fibrosis

**DOI:** 10.1371/journal.pone.0107175

**Published:** 2014-09-12

**Authors:** Graeme C. Elliott, Rockesh Gurtu, Charles McCollum, William G. Newman, Tao Wang

**Affiliations:** 1 Centre for Genomic Medicine, Institute of Human Development, Faculty of Medical and Human Sciences, The University of Manchester, Manchester, United Kingdom; 2 Academic Surgery Unit, Education and Research Centre, University Hospital of South Manchester, Manchester, United Kingdom; 3 Centre for Genomic Medicine, Central Manchester University Hospitals NHS Foundation Trust, Manchester, United Kingdom; William Harvey Research Institute, Barts and The London School of Medicine and Dentistry, Queen Mary University of London, United Kingdom

## Abstract

Patent foramen ovale (PFO) is an atrial septal deformity present in around 25% of the general population. PFO is associated with major causes of morbidity, including stroke and migraine. PFO appears to be heritable but genes involved in the closure of foramen ovale have not been identified. The aim of this study is to determine molecular pathways and genes that are responsible to the postnatal closure of the foramen ovale. Using Sprague-Dawley rat hearts as a model we analysed the dynamic histological changes and gene expressions at the foramen ovale region between embryonic day 20 and postnatal day 7. We observed a gradual loss of the endothelial marker PECAM1, an upregulation of the mesenchymal marker vimentin and α-smooth muscle actin, the elevation of the transcription factor Snail, and an increase of fibroblast activation protein (FAP) in the foramen ovale region as well as the deposition of collagen-rich connective tissues at the closed foramen ovale, suggesting endothelial-to-mesenchymal transition (EndMT) occurring during foramen ovale closure which leads to fibrosis. In addition, Notch1 and Notch3 receptors, Notch ligand Jagged1 and Notch effector HRT1 were highly expressed in the endocardium of the foramen ovale region during EndMT. Activation of Notch3 alone in an endothelial cell culture model was able to drive EndMT and transform endothelial cells to mesenchymal phenotype. Our data demonstrate for the first time that FO closure is a process of EndMT-mediated fibrosis, and Notch signalling is an important player participating in this process. Elucidation of the molecular mechanisms of the closure of foramen ovale informs the pathogenesis of PFO and may provide potential options for screening and prevention of PFO related conditions.

## Introduction

The foramen ovale (FO) is an opening in the septum between the left and right atria of the fetal heart. It is a flap-valve like structure that allows oxygenated blood from the placenta to flow from the right to left atrium into the fetal systemic circulation, bypassing the non-functional fetal lungs. At birth, lung function and pulmonary circulation is established by the first breath. This significantly decreases the pulmonary vascular resistance and the pressure in the right heart forcing the flap-valve against the FO opening, resulting in the functional closure of the FO. Tissue fusion is then believed to occur leading to FO closure. This closure fails in approximately 25% of individuals where the FO persists into adulthood as a patent foramen ovale (PFO) [1].

Although PFO was previously considered to be benign, there is growing awareness of the potentially pathological sequelae of PFO in the last two decades. A significantly high prevalence of PFO has been noted in cryptogenic stroke in young adults (55–60%) [2], migraine with aura (41–53%) [3], sudden deafness (48%) [4], and decompression sickness in divers (68%) [5]. It is thought that PFO allows paradoxical embolization of venous thrombi directly into the arterial circulation [6]. PFO may also allow paradoxical passage of vasoactive substances such as in the case of carcinoid heart disease in which the incidence of PFO is as high as 59% [7]. The right-to-left shunt through a PFO bypasses the lungs avoiding the inactivation of carcinoid tissue-released serotonin. PFO also contributes to the onset of platypnea orthodoxia [8]. Percutaneous closure of PFO may be indicated for the prevention of these PFO-related complications [8].

The reasons why the FO remains patent in some individuals is poorly understood. Accumulating evidence suggests that genetic factors contribute to the persistence of PFO and other defects of atrial septation, including atrial septal defects (ASD) [9], [10], [11]. A number of studies have shown that ASD exhibits autosomal dominant inheritance, and a list of causative genes encoding cardiac transcription factors and their targets (*NKX2-5* [12], *TBX5* [13], *GATA4* [10], *MYH6* [14], *ACTC* [15], *TBX20* [16]) have been identified. Variation in these genes or their expression may contribute to the development of PFO. To date a number of candidate gene case-control association studies, including the two pilot studies by our own group on *NKX2-5* and *GATA4*, have not demonstrated any significant relationship between PFO and variants in these cardiac septum-developmental genes [17], [18]. This highlights the poor understanding of PFO development and the natural process of FO closure.

The septum primum (SP) grows down from the roof of the atria at the sixth week of human embryonic development. The SP is led by a cap of mesenchymal tissue derived from the endocardial cushion [19]. This mesenchymal cap fuses with the endocardial cushion to anchor the SP to the base of atria [20]. While this occurs, apoptosis creates an opening in the SP near the roof of the atria to maintain the blood flow between the right and left atria. The septum secundum (SS) then forms as an infolding of the atrial roof and grows down into the right atrium [21]. This forms a thick ridge of tissue for the flap-like SP to rest against when the FO closes at birth. The completed FO acts a one-way valve-like structure; however, the morphological changes after birth during the postnatal closure of FO have not been well documented. In mice, the FO is partially closed by postnatal day P3 and fully closed by P7 [22]; in rats, the FO is closed by P3 with rapid thickening of the SP seen at P2 [23]; in human adults, fibrotic tissue has been observed around the SP [24], [25]. However, the dynamic histological alterations in the process of FO closure are not well recorded; the underlying molecular mechanisms that drive FO fusion are largely unknown.

Endothelial to mesenchymal transition (EndMT) is an essential process that occurs during embryonic development of the heart [26]. Mesenchymal cell generation through EndMT from the endocardial cushion at human embryo day 32 is a key process contributing to the formation of cardiac septa and valves [27]. However, it is unknown whether EndMT is also involved in the closure of the FO. Essential signalling that regulates EndMT includes TGF-β, Wnt and Notch pathways, of which Notch signalling is a critical player that drives early EndMT by activating Snail, the prototypical EMT-inducing transcription factor [26], [28], [29], [30].

Proteins of the Notch family are cell surface receptors. Notch signalling is responsible for cell fate determination through transducing signals from the neighbour cells [31]. There are four types of Notch receptor (Notch 1–4) and five ligands (Delta-like-1, -3 and -4, and Jagged-1 and -2) found in mammals. Ligand binding triggers proteolysis cleavage of Notch receptor to release the intracellular domain of Notch (NICD) which translocates into the nucleus where it binds to and switches the transcription suppressor RBP-Jκ into a transcriptional activator, regulating Notch target gene expression. Interestingly, a high prevalence of PFO is recorded in patients with CADASIL (Cerebral Autosomal Dominant Arteriopathy with Subcortical Infarcts and Leukoencephalopathy) [32], an inherited stroke syndrome due to mutations in *NOTCH3*. A case report of multiple family members with CADASIL revealed an 80% prevalence of PFO [33], suggesting a potential role of Notch3 in the closure of the FO.

In this study, we used the Sprague-Dawley rat heart as a model to systemically examined the morphological changes of the atrial septum at the age of very late embryonic development day 20 (E20) and postnatal day 1–7 (P1–P7) which spans the entire course of FO closure and beyond. In conjunction with gene expression and cell culture experiments, we describe for the first time that EndMT is the underlying molecular mechanism for FO closure which leads to regional fibrosis with the possible involvement of the Notch signalling. Our finding contributes to knowledge on neonatal heart development, a necessary step towards fully understanding the pathogenesis of PFO and defining genetic biomarkers to aid clinical diagnosis and treatment.

## Materials and Methods

### Tissue preparation and histological staining

Sprague-Dawley rats were sacrificed using cervical dislocation as per schedule 1 procedure in the Biological Service Facility in the University of Manchester according to guidance in the UK Home Office Animals (Scientific Procedures) Act 1986 under licence PPL 40/3409. Whole hearts were dissected from adult rats and neonates ages between P1 and P7. E20 hearts obtained by dissecting fetal rats from the womb before removing the heart were fixed in formalin for 24 hours before processing and embedding in wax. Serial 7 µm wax sections of the hearts from each age group were stained with haematoxylin and eosin (H&E). Adult heart sections were stained with Martius Scarlet Blue (MSB). Images were taken using an Axioskop upright microscope with the Axiocam colour CCD camera.

### Immunohistochemistry

Sections from rat hearts aged E20, P1, P3 and P7 were immunohistologically stained and dewaxed in xylene and rehydrated in a decreasing ethanol series. Antigen retrieval was performed in 10 mM citric acid, pH 6, at 97°C for 30 minutes. For immunofluorescent staining, 0.25% ammonium chloride was added to the section to block autofluorescence; for DAB staining, 3% hydrogen peroxide was added to the sections to block endogenous peroxide activity. The sections were then permeabilised for 10 minutes in 0.1% triton X-100 in phosphate buffer saline (PBS) followed by incubation with 5% bovine serum albumin (BSA) in PBS for 60 minutes to block the non-specific binding sites. The sections were then incubated with primary antibodies with appropriate dilutions (see below), washed with PBS, and incubated with secondary antibodies listed below for 1 hour at room temperature. After final washes, the fluorescent-stained samples were mounted with VECTASHIELD Mounting Medium containing DAPI (Vector Laboratories); the colorimetric-stained samples were counterstained with haematoxylin and mounted with Pertex mounting medium.

Primary antibodies used were acquired from Santa Cruz Santa (Cruz Biotechnology, Inc): PCNA (FL-261, 1∶100 dilution), SNAI1 (H-130, 1∶200 dilution), PECAM1 (M-20, 1∶25 dilution), Vimentin (V9, 1∶100 dilution), Notch3 (M-134, 1∶400 for DAB and 1.25 for immunofluorescence), Notch1 (1∶100 dilution), Jagged1 (C-20, 1∶200 dilution) and HRT1 (H-120, 1∶200 dilution). The α-smooth muscle actin antibody (1∶200 dilution) was acquired from Abcam plc, UK. For immunofluorescent staining, secondary antibodies were purchased from Invitrogen: donkey anti-mouse IgG AlexaFluor 594 (1∶200 dilution), donkey anti-goat IgG AlexaFluor 594 (1∶200 dilution), rabbit anti-goat IgG AlexaFluor 488 (1∶200 dilution), and donkey anti-rabbit IgG AlexaFluor 488 (1∶1000 dilution). For colorimetric staining, a DAB (3, 3′-diaminobenzidine) substrate kit (SK-4100, Vector laboratories) was used with HRP conjugated polyclonal goat anti-rabbit and rabbit anti-goat IgGs (Dako, 1∶100 dilution). Images were taken using Olympus BX51 upright fluorescent microscope or Axioskop upright microscope with the Axiocam colour CCD camera.

### Laser microdissection and qRT-PCR

The entire hearts dissected from E20, P1, P2, P3 and P7 rats are immediately snap frozen in isopentane on dry ice, and then embedded in OCT compound and stored at −80°C. The hearts are sectioned at 50 µm thickness and mounted onto RNase Zap treated PEN Membrane Slides (Leica Microsystems UK) for laser dissection. The foramen ovale region were dissected from the tissue sections under microscope and deposited into lysis buffer containing β-Mercaptoethanol ready for total RNA extraction using the Qiagen RNeasy Micro kit. cDNA was synthesised and amplified using the Ovation WTA System V2 kit (Nugen). This was followed by real time quantitative PCR (qPCR) using SYBR Green Master Mix (Applied Biosystems). Notch1 qPCR primers: forward 5′CTGTTGTGCTCCTGAAGAAC 3′; reverse 5′GTCCATGTGATCCGTGATGT 3′. Notch3 qPCR primers are: forward 5′CCAGATTCTCATCAGGAACC3′; reverse 5′CTGTCCTGCATGTCCTTGTT 3′. Jagged1 qPCR primers are: forward 5′CACATTTGCAGCGAATTGAG 3′; reverse 5′CAGAGGAACCAGGAAATCTG 3′. HRT1 qPCR primers: forward 5′GACTACAGCTCCTCTGATAG 3′; reverse 5′CAGGTGATCCACAGTCATCT 3′. qPCR primers for fibroblast activation protein are: 5′AGCCATATGGGGATGGTCCT 3′; reverse 5′TGTTGGGAGGCCCATGAATC 3′.

### Cell culture and Immunocytochemistry

Human coronary artery endothelial cells (hCAECs, PromoCell, C-12221) were cultured in endothelial cell growth medium MV (PromoCell, C-22020) on coverslips in 6-well plates (1×10^5^ cells per well). After 24 hours, the culture medium was removed and cells were incubated with 300 µl adenovirus which contains the intracellular domain of Notch3 (N3ICD), or the empty adenovirus vector (Control), in PBS at a MOI 50 for 45 minutes. The virus solution were then removed and replaced by 2 ml endothelial cell growth medium MV. The cells were cultured for a further 2 days or 7 days before examination. Immunofluorescent staining was as previously described [34]; briefly, the cells were fixed for 20 minutes in 4% paraformaldehyde in PBS followed by incubation with primary antibodies in PBS containing 5% fatal calf serum (FCS) for 1 hour. After washing x4 with 5% FCS in PBS for 5 minutes, the samples were then incubated with AlexaFluor-conjugated secondary antibodies. After final wash with 5% FCS in PBS, the coverslips were mounted on microscope slides with VECTASHIELD Mounting Medium containing DAPI (Vector Laboratories). Images were taken using Olympus BX51 upright fluorescent microscope.

Primary antibodies used for immunofluorescent staining were from Santa Cruz: SNAI1 (H-130, 2∶25 dilution), PECAM1 (M-20, 1∶25 dilution), Vimentin (V9, 1∶200 dilution), and Notch3 (M-20 and M-134, 1∶25 dilution). Secondary antibodies used were purchased from Invitrogen: donkey anti-mouse IgG AlexaFluor 594 (1∶200 dilution), donkey anti-goat IgG AlexaFluor 594 (1∶200 dilution), rabbit anti-goat IgG AlexaFluor 488 (1∶200 dilution), and donkey anti-rabbit IgG AlexaFluor 488 (1∶200 dilution).

### Western Blotting

Western blotting was also carried out as described previously [34]. hCAECs growing in 6-well plates were washed twice with PBS. Whole cell lysates were prepared by scraping the cells in 300 µl in Leammli buffer (62.5 mM, Tris–HCl, pH 6.8, 25% glycerol, 2% SDS, 5% β-mercaptoethanol and bromophenol blue) and denatured at 100°C for 5 minutes. Cell lysates were subjected to 6% or 10% SDS-polyacrylamide gel electrophoresis and the proteins were transferred onto nitrocellulose membrane followed by incubation with Primary antibodies (1∶200 dilutions) and HRP conjugated secondary antibodies (1∶1000 dilutions, DACO). Signals were visualized using enhanced chemiluminescence reagent (ECL, GE Healthcare, Life Sciences) and exposed onto ECL Hyperfilm (GE Healthcare, Life Sciences). Protein loading was normalized by stripping off the antibodies and reprobing the blot with anti-β-actin antibodies.

### Statistics

Data are presented as mean ± SEM. Differences between groups were analysed using Student's t-tests with p<0.05 considered statistically significant.

## Results

### Morphological changes during FO closure

**Figure 1:**
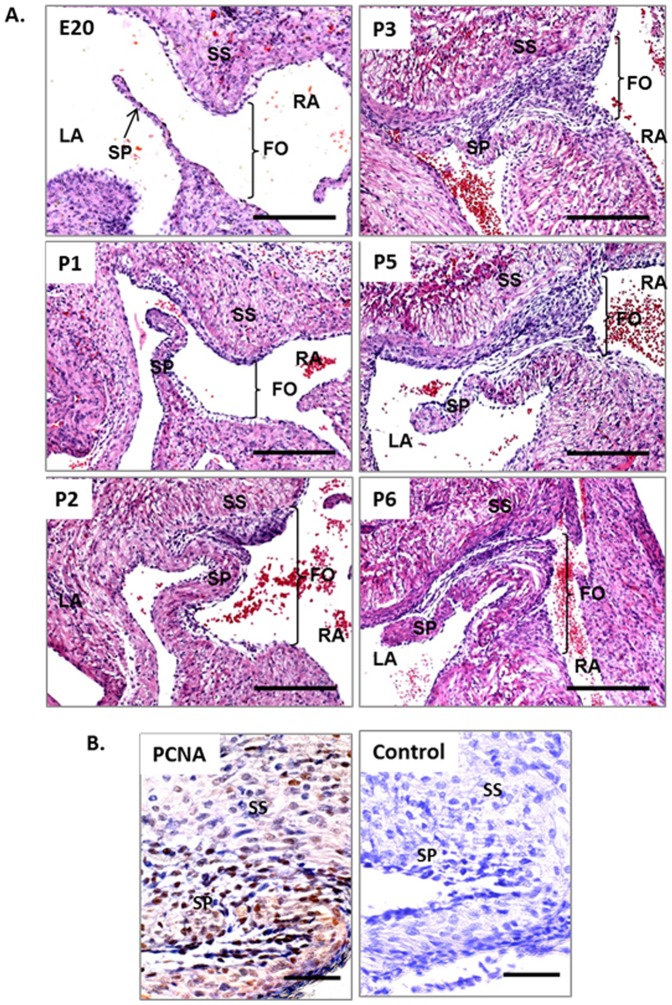
Selected tissue sections of the FO region showing FO closure in the fetal and neonatal rat heart. **A**. Closure and fusion of the FO during E20–P6. **B**. Immunohistochemical staining for nuclear cell proliferation marker PCNA (brown) at P2. Both PCNA and Control sections were counterstained with haematoxylin showing blue nuclei. Scale bars = 200 µm in A; scale bars = 50 µm in B. RA, right atrium; LA, left atrium; SP, septum primum; SS, septum secundum; FO, foramen ovale.

To examine the changes in FO morphology during FO closure, tissue sections from E20-P7 Sprague Dawley rat hearts were isolated and sections were cut from dorsal to ventral across the coronal plane of the heart. The H&E stained tissue sections in Figure 1 summarise the dynamic closure of the FO.

At E20 the SP was a thin flap of tissue only a few cells thick. The SS could be observed growing down from the roof of the atrium. On the first day after birth (P1) the SP became significantly thicker with a clear centre of myocardium, which was covered with a more prominent subendocardial layer. The endocardial regions around the SS and the right surface of SP gradually enlarged form P1 onwards. Immunohistochemical labelling of cell proliferation marker PCNA at P2 showed strong staining in the endocardial region where the SP and SS meet (Figure 1B), indicating active cell proliferation occurring. The FO was still open allowing possible blood flow at P2. By P3 the thickened endocardial regions of SS and the right side of the SP had met merged and closed the FO (Figure 1A). Despite this closure, the SP has not fully fused to the SS. Figure 1A shows that the distal region of the SP flap was still free at P5. It was not until P6 that SP appeared fully fused completing the atrial septum. This suggests that the initial closure of FO is due to the merger of the endocardial layers of the SP and SS and cell proliferation emanating from the endocardium of this region.

### Collagen-rich connective tissue is present at fused FO region

**Figure 2:**
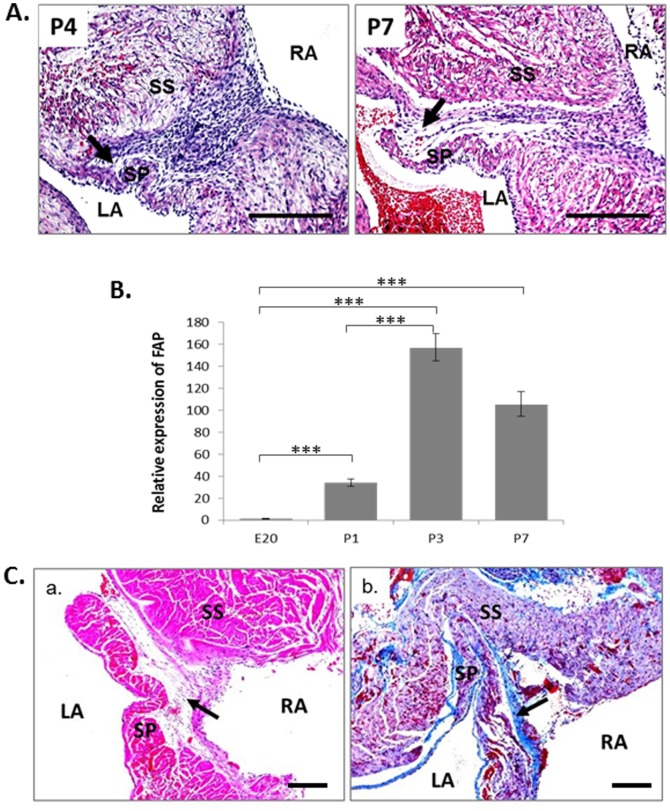
Evidence of fibrosis during and after FO closure in rat heart. **A**. H&E staining of FO region in neonates P4 and P7. Reduced cell density is shown by the arrows. Scale bars = 200. **B**. FAP mRNA expression during FO closure as determined by qRT-PCR. ***p<0.001, n = 3. **C**. Evidence of fibrosis in the remainder of FO region in adult rat hearts. a. H&E staining; b. Martius Scarlet Blue (MSB) staining. Scale bars = 500 µm. RA, right atrium; LA, left atrium; SP, septum primum; SS, septum secundum.

After the initial fusion of the FO, further morphological changes to the atrial septum included a decrease in cytoplasmic staining and a gradual decline in nuclear density in this region from P4 onwards (Figure 2A). These changes were more prominent at P7 (Figure 2A), indicating an increase in extracellular matrix (ECM) in the region, suggesting active fibrosis.

Microarray analysis was performed on RNA samples extracted by laser microdissection from FO region of E20-P7 Sprague Dawley rat heart tissues sections. The transcription of four fibrosis-related genes were up-regulated, among which the fibroblast activation protein (FAP) was consistently raised after birth at P1 and peaked on day 3 as determined by qRT-PCR (Figure 2B).

To further demonstrate the cellular changes in this region, the mature atrial septum was examined in adult Sprague-Dawley rat hearts (Figure 2C). The remnants of the SP and SS could still be seen in the adult atrial septum. The tissue closing the FO showed a further decrease in nuclear density and less cytoplasmic staining than in the neonatal heart (Figure 2C–a). To search for evidence of connective tissue in the region a Martius Scarlet Blue stain was used (Figure 2C–b). The light blue staining indicated collagen was present throughout the closed FO region. The light red staining showed cardiac muscle tissue present in the remnants of the SP and SS, but not in the tissue fusing them together. This suggests that the FO was eventually closed by collagen-rich connective tissue.

### EndMT is occurring during FO closure

During fibrosis, collagen is synthesized by fibroblasts, a mesenchymal cell type that may be transformed from the endocardium via EndMT [35]. During EndMT, endothelial cells gradually gain a mesenchymal phenotype as indicated by the loss of endothelial markers such as PECAM1 and the increased expression of mesenchymal markers, like vimentin. To investigate if EndMT is involved in FO closure, the protein expression patterns of PECAM1 and vimentin as well as the transcription factor Snail, the key mediator of EndMT, were determined in tissue sections of rat hearts during FO closure.

**Figure 3:**
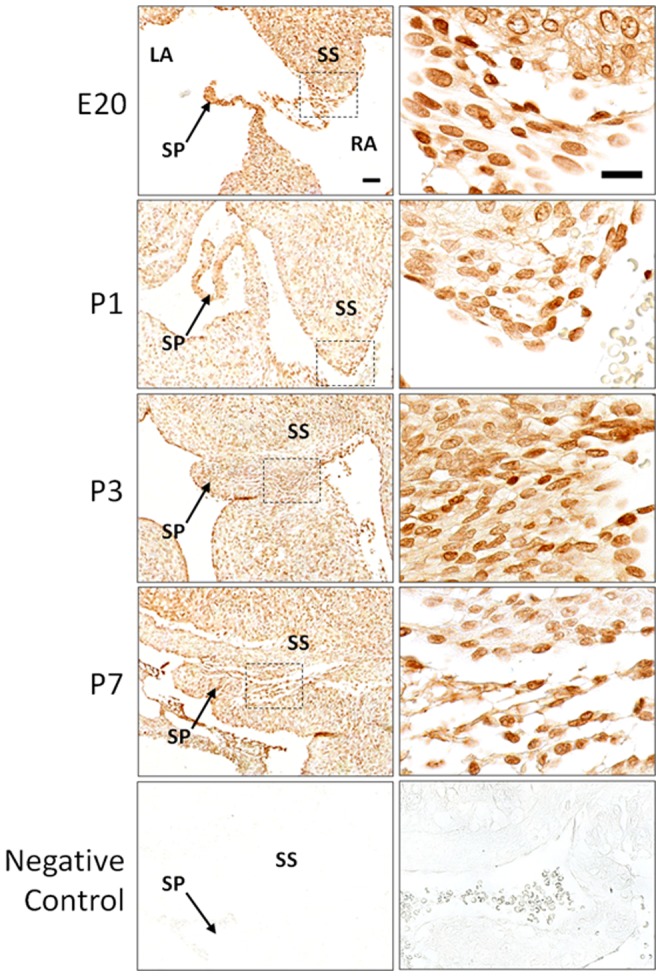
Immunohistochemistry staining for Snail in rat heart tissue sections during FO closure. Positive signals are brown colour by DAB staining. Scale bars = 50 µm. Negative control: sections were stained with secondary antibodies only.

**Figure 4:**
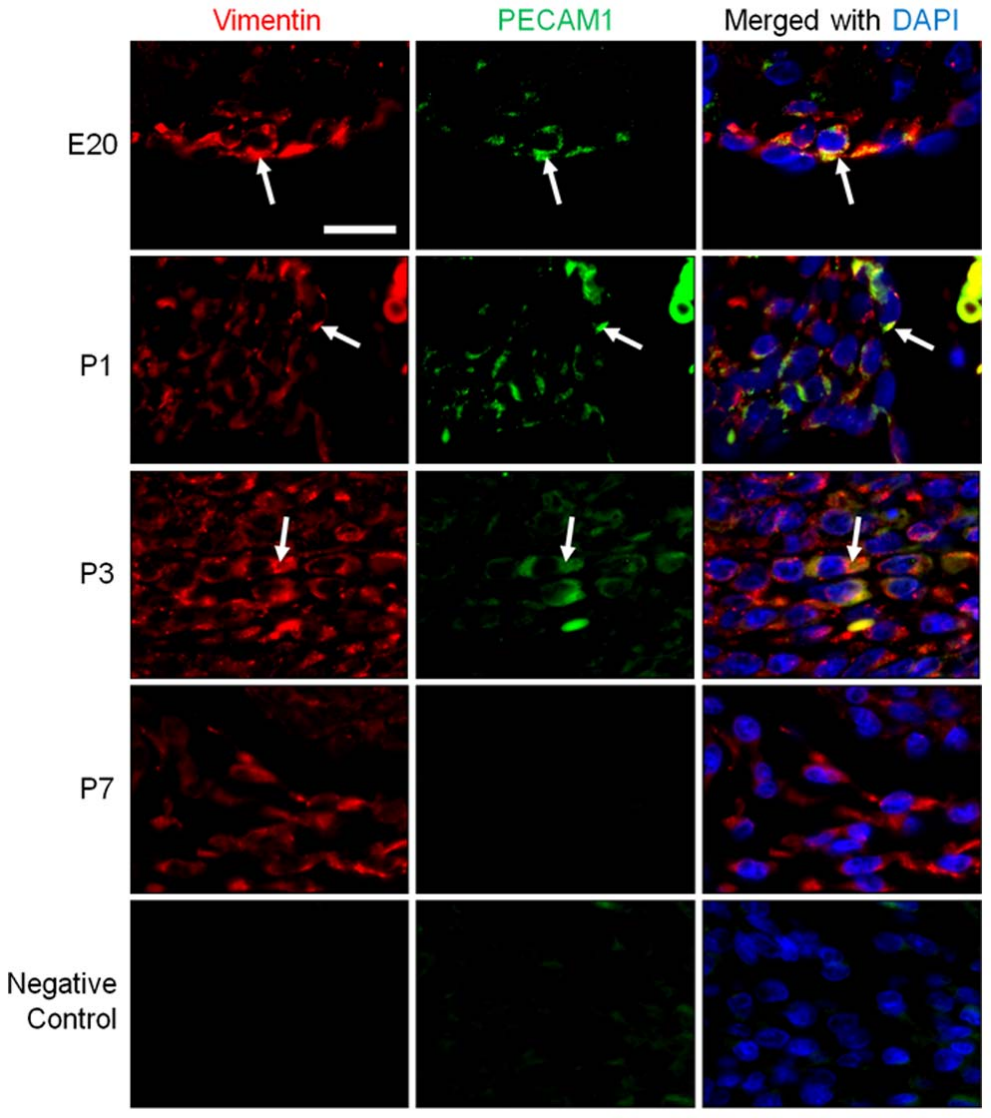
Immunohistochemistry staining for vimentin and PECAM1 in rat heart tissue sections during FO closure. Double immunefluorescent staining was performed for vimentin (red) and PECAM1 (green). Scale bars = 50 µm. Nuclei were counterstained with DAPI (blue). Arrows indicate cells expressing both PECAM1 and vimentin. Negative control: sections were stained with secondary antibodies only.

At E20, the nuclear expression of Snail could be detected in the endocardial region of the SP and SS (Figure 3), suggesting that EndMT is already occurring in the endocardium at this early stage before birth. Using fluorescent double staining, the endothelial marker PECAM1 and mesenchymal marker vimentin were both found in the same cells (Figure 4, arrows), supporting an endocardial to mesenchymal transition occurring in these cells. The EndMT at this developmental stage likely contributes to the prenatal growth of SP and SS.

At P1 the nuclear expression of Snail was present throughout the thickened endocardial layer of the SP and SS (Figure 3). The expression of PECAM1 was observed mainly near the surface of this thickened tissue and decreased deeper into the myocardium (Figure 4), suggesting a gradual loss of endothelial phenotype. Vimentin expression was present throughout the thickening endocardial region, and co-exists with PECAM1 in the same cells (Figure 4, arrows), suggesting that cell growth and thickening of the endocardial region were driven by EndMT.

At P3 vimentin was expressed throughout the tissue fusing the FO. PECAM1 was restricted to cells running through the centre of the tissue in the FO region where the SP and SS meet (Figure 4, arrow), indicating the majority of the tissue closing the FO was of a mesenchymal phenotype.

By P7 the expression of PECAM1 disappeared in the fused FO region while vimentin was still present throughout (Figure 4), suggesting the completion of EndMT. In the ECM-rich areas, nuclear Snail expression was continually observed (Figure 3). This perhaps indicated a further role for Snail in mesenchymal cells.

Collectively, the results suggest that EndMT occurs in the endocardial regions of the FO before birth and is highly activated throughout the course of FO fusion. This allows the production of mesenchymal tissue with increased ECM deposition which eventually closes the FO.

### Notch signalling is active during FO closure

Notch signalling has been reported to be a critical regulator of EndMT by upregulating Snail [36] and plays an important role in cardiac development [30]. Increased PFO prevalence was observed in patients with CADASIL, a condition caused by germline *NOTCH3* mutations, suggesting a potential role of Notch3 in FO closure. We therefore examined the expression of Notch receptor related genes during FO closure.

**Figure 5:**
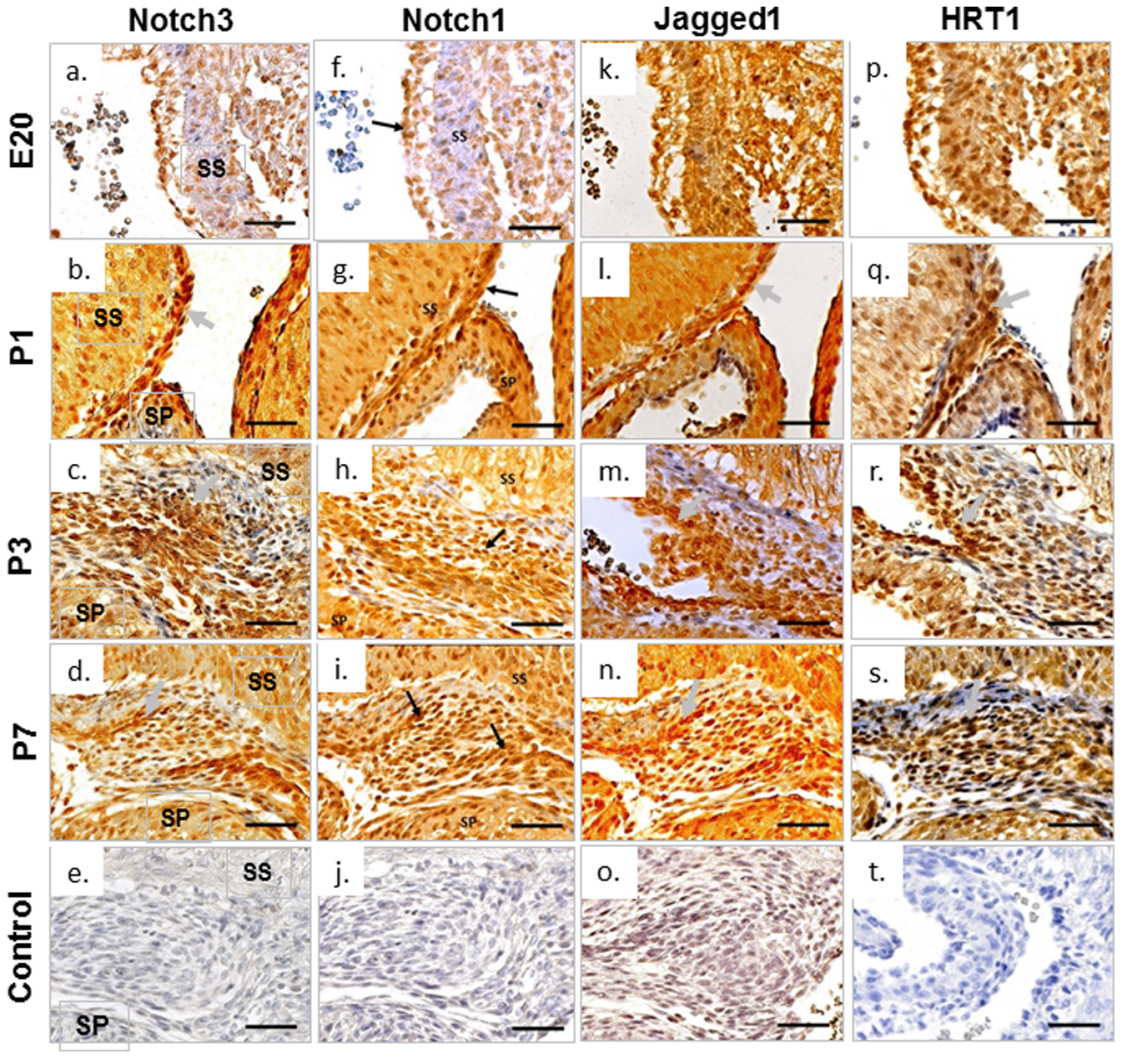
Immunohistochemistry staining of Notch pathway proteins during FO closure in rat heart. Tissue sections of rat FO region were immunestained with Notch1, Notch3, Jagged1 and HRT1 using specific antibodies and visualised by DAB detection kit (brown). All sections were nuclear counterstained with haematoxylin (blue). Scale bars = 200 µm. SP, septum primum; SS, septum secundum. Arrows indicate highly expressed proteins.

At E20 the expression of Notch1 and Notch3 could be seen in both myocardium and endocardium of the FO region (Figure 5-a,f). At P1, Notch3 appeared mainly located in the endocardial region (Figure 5-b). By P3 its expression was restricted to the centre region of FO (Figure 5-c) where the endocardium of SS and SP meet and where EndMT is occurring as observed in Figure 3 and 4. Notch3 was still observed in the mesenchymal tissue at the centre region of FO by P7 (Figure 5-d). Notch1 had a similar expression pattern to Notch3 at P1, P3 and P7, but its location was more diffuse across the endocardium and myocardium compared to Notch3. Both Notch1 and Notch3 displayed a clear pattern of nuclear staining in addition to cytoplasm signals, indicating activation of the receptors in these cells.

To further investigate the activity of Notch signalling, the Notch ligand Jagged1 and downstream target HRT1 were also assessed via immunohistochemistry. At E20, both Jagged1 and HRT1 were detected throughout myocardium and endocardium (Figure 5-k, p), suggesting ligand-mediated activation of Notch signalling occurring in the FO region which is in line with the expression of Notch1 and Notch3. By P1, a clear pattern of endocardium location could be observed for both Jagged1 and HRT1 (Figure 5-l, q). By P3, both proteins were highly restricted to the endocardium of FO region and less dense deeper into the mesenchyme adjacent to the myocardium (Figure 5-m, r). By P7, Jagged1 and HRT1 could still be detected in the FO region (Figure 5-n, s).

**Figure 6:**
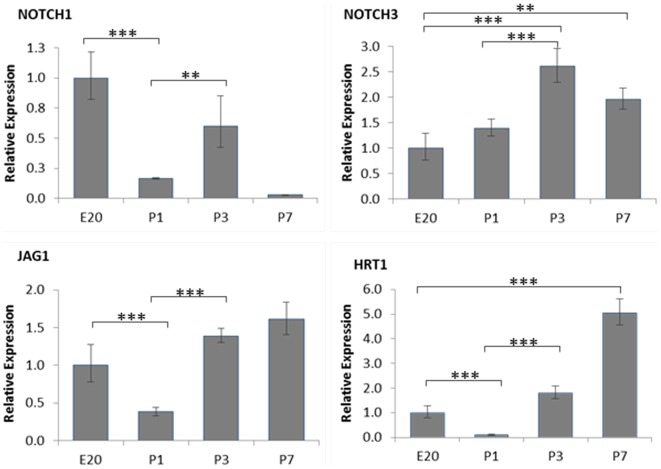
The mRNA expression of Notch1, Notch3, Jagged1 and HRT1 in the FO region of rat heart. Total RNA of FO region was extracted from tissue samples obtained by laser microdissection and subjected to qRT-PCR. Results are presented as relative expression in comparison to E20. **p<0.01, ***p<0.001, n = 3.

To quantify the mRNA expression of the Notch signalling pathway components, qRT-PCR was performed on RNA extracted from the tissue sections of FO region obtained by laser microdissection. A significant increase in Notch3 protein level was observed from E20, peaking at P3, and dropping slightly by P7 (Figure 6). The total expression of Notch1, Jagged1 and HRT1 were unexpectedly lower at P1 in comparison to E20 (Figure 6). Despite this, Notch1, Jagged1 and HRT1 were all increased postnatally during the course of FO closure from P1 to P3 (Figure 6).

### Notch3 activation alone induces EndMT in endothelial cells

Previous studies showed that Notch1 activation could induce EndMT during cardiac development and oncogenic transformation [30]. However, it has not been demonstrated whether Notch3 could directly and independently drive EndMT in endothelial cells. Given the specific location of Notch3 in the endocardium of the FO region and its significant increase in expression during FO closure, we proceeded to test the ability of Notch3 alone to induce EndMT in endothelial cells. Due to the fact that the endothelium of the coronary arteries are generated from endocardial cells during embryonic development [37], human coronary artery endothelial cells were used as a model for the activation of Notch3 signalling using adenovirus-mediated overexpression of the constitutive active form of Notch3, N3ICD.

**Figure 7:**
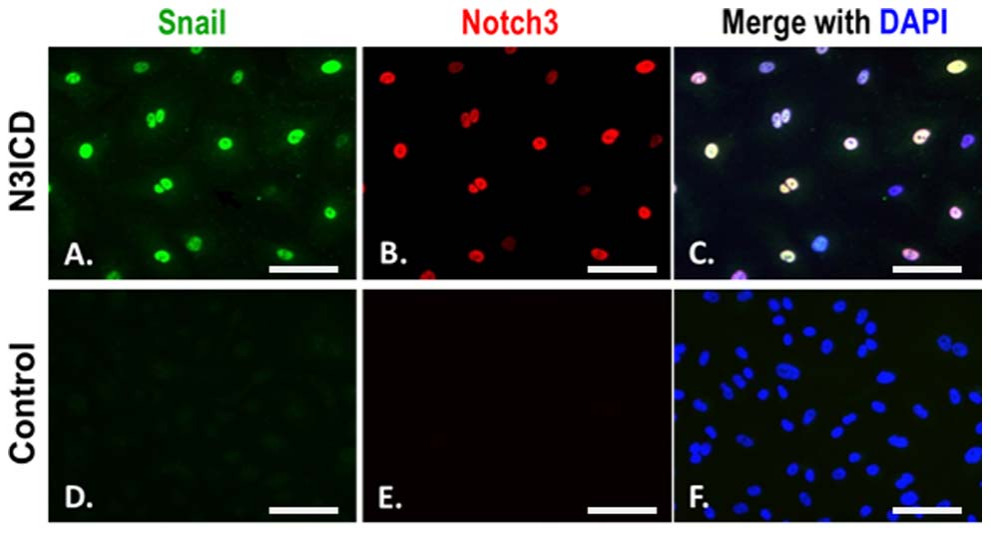
Activation of Notch3 in endothelial cells induces nuclear Snail expression. hCAECs were infected by adenovirus-N3ICD (A–C) or adenovirus vector-only (D–F). 48 hours after the infection the cells were double stained for Notch3 (red) and Snail (green). **C** and **F**, merged image with DAPI staining of the nuclei. Scale bar = 50 µm.

Upon Notch3 activation, the nuclear expression of Snail was clearly induced and specifically restricted to endothelial cells overexpressing N3ICD. The induction of Snail expression was not seen in cells overexpressing the adenovirus control vectors (Figure 7), supporting the role of Notch3 in the initiation of EndMT.

**Figure 8:**
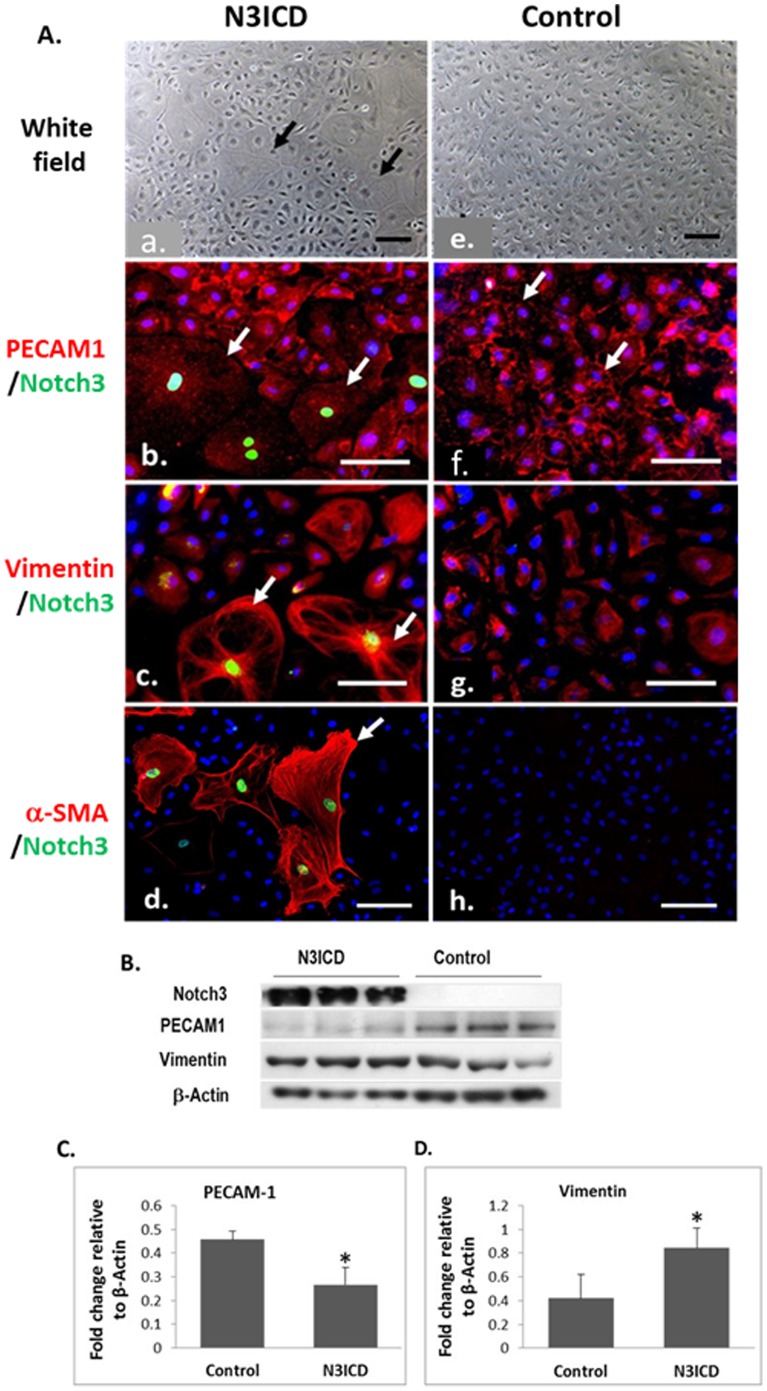
Activation of Notch3 in endothelial cells induces cellular changes typical of EndMT. **A**, Changes in cell morphology 7 days after infection (A-a), and the expression of vimentin and PECAM1 in N3ICD infected cells (A-b and A-c). Arrows in A-b indicate loss of membrane staining of PECAM1 in Notch3 positive cells; and arrows in A-f indicate membrane PECAM1. Arrows in A-c indicate cells exhibiting cell spreading. **B**, Western blotting for mesenchymal marker (vimentin) and endothelial markers (PECAM1) with β-Actin as a protein loading control. **C** & **D**, Quantitation of the protein expressions of PECAM1 (C) and vimentin (D). Data presented as the ratio to β-Actin, mean ± SE. *p<0.05, n = 3. Scale bar = 50 µm.

Seven days after N3ICD overexpression, the endothelial cells showed significant morphological changes. These cells were enlarged with increased cell spreading and an irregular shape (Figure 8A-a, arrows). The control endothelial cells showed the presence of PECAM1 around the cell membranes and at cell-cell contacts (Figure 8A-e, arrows), while the N3ICD positive cells showed decreased PECAM1 levels with absence at the membrane (Figure 8A-b, arrows). The Notch 3 induced decrease in PECAM1 expression was confirmed by western blotting (Figure 8B, C).

In the N3ICD overexpressing endothelial cells, the mesenchyme marker vimentin was reorganised with more defined filaments extending towards the cell membrane (Figure 8A-c, arrows), as seen in fibroblasts. Also, vimentin filaments were observed near the edges of the cells running parallel to the cell membrane (Figure 8A-c). Immunoblotting analysis showed a significant increase in vimentin level in N3ICD infected cells (Figure 8B, D). An additional mesenchymal marker, α-smooth muscle actin (α-SMA), was also induced by Notch3 activation, which is accompanied by the morphological changes of the endothelial cells to a mesenchymal appearance (Figure 8A-d). We randomly counted 100 N3ICD overexpressing cells and found 73 cells were α-SMA positive, suggesting a transdifferentiation efficiency of around 70% was achieved. There were no α-SMA positive cells found in the non-N3ICD overexpressing control endothelial cell population (Figure 8A-h).

## Discussion

By analysing tissue sections at different time intervals before and during FO closure in rat hearts, we have illustrated novel insight of the histological changes seen during FO closure. Using a combination of immunohistological methods and endothelial cell culture, we were able to demonstrate that FO closure is driven by EndMT mediated fibrosis with the possible involvement of Notch signalling.

In Sprague-Dawley rats, the FO was closed between 2 and 3 days after birth, supporting a previous study in Wistar rats [23]. Despite the FO communication being permanently closed, the tip of the SP remained mobile, forming a pouch-like structure until the complete fusion of the SP to the SS between P5 and P6. The remnant of the pouch-like structure, if persisting into adult life, could have pathological implications by harbouring thrombi, which increases the risk of stroke. Interestingly, the pouch-like structure has recently been reported in clinical cases and autopsied human hearts with evidence of thrombus formation [38].

A gradual but dramatic thickening of the endocardial region in SS and along the right side of SP was observed from P1 to P3. The presence of nuclear Snail and the loss of the endothelial marker PECAM1, along with increased levels of the mesenchymal marker vimentin and α-SMA, suggest EndMT is occurring during FO closure. Down-regulation of PECAM1 indicates a loss of cell-cell connections in addition to the loss of endothelial cell characteristics, which facilitates the migration of newly formed mesenchyme cells through EndMT. It is known that EndMT is an important mechanism involved in endocardial cushion formation, heart septation and valve development [26]. Our data suggest that EndMT is also important for the postnatal FO closure.

After the initial closure of the FO at P3, we observed an increase of ECM in the FO region, resulting in a mature adult atrial septum with the FO replaced by connective tissue. Indeed, previous histological analysis of human adult hearts has noted fibrous tissue around the remainder of the FO in the atrial septum [24], [25]. Furthermore, we observed significant increase in the expression of FAP in the FO region after birth. The gene FAP encodes a homodimeric integral membrane gelatinase belonging to the serine protease family [39]. It is not normally expressed in healthy adult human tissues but is expressed during early development in mesenchymal tissue remodelling [40]. FAP expression is thought to control fibroblast growth or epithelial-mesenchymal interactions [39]. In adults, FAP is upregulated during tissue repair [41], pathological fibrosis [42], [43], or in tumors [44], [45]. The significant increase in the expression of FAP from P1 to P7 suggests active fibrosis is occurring during the course of FO closure. EndMT is known to be an important source of fibroblasts which could lead to pathological cardiac fibrosis [46]. Our data suggest that EndMT-mediated fibrosis is also a physiological process which could be essential in postnatal heart development and remodelling. Fibrosis may also contribute to the rigidity of the adult atrial septum. Failure of this process may lead to atrial septal aneurysms (ASA) which are strongly associated with PFO in man [47].

In the study, we also undertook preliminary exploration of the role of Notch signalling in FO closure. It is known that Notch signalling is important in regulating EndMT in heart development, particularly the role of Notch1 in the development of the atrioventricular canal, endocardial cushions and valves [48]. However, the function of Notch3 in this process has not been characterised. We used immunohistological staining to show that both Notch1 and Notch3 were clearly present in the FO region during the course of FO closure. This is accompanied by the presence of the Notch ligand, Jagged1 and Notch target gene, HRT1, suggesting the possibility of ligand mediated activation of Notch signalling. Notch1, Notch3, Jagged1 and HRT1 all displayed a clear pattern of endocardial staining along the meeting front of SS and SP, and the interface where SP and SS meet and fuse, suggesting the possible involvement of Notch signalling in driving the EndMT process which leads to FO closure. Interestingly, compared to Notch1, the regional endocardial pattern of Notch3 was clearer and the overall expression of Notch3 mRNA steadily increased after birth, supporting the active involvement of Notch3 in the EndMT during FO closure. Among the four Notch receptor subtypes, Notch3 is considered to be arterial specific and plays an important role in the maturation of arterial smooth muscle cells (SMCs) [49]. Unlike Notch1 and Notch2, absence of which are embryonic lethal [50], Notch3 knockout mice are vital and fertile [49], suggesting a role for Notch3 in the late stage of embryonic development or an important postnatal function. Consistent with this hypothesis, individuals with CADASIL show characteristic pathological changes of SMC degeneration in small arteries in adults. We found that the total expression of Notch1, Jagged1 and HRT1 were unexpectedly lower at P1 in comparison to E20 (Figure 6). This may reflect a high level of ligand mediated Notch1 activity in the prenatal heart. However, the postnatal increases of Notch1, Jagged1 and HRT1 from P1 to P3 further support the activation of Notch signalling during the course of FO closure.

To demonstrate the direct role of Notch3 in inducing EndMT, we activated Notch3 in human coronary endothelial cells, a descendant cell type of endocardium [37], and observed increased Snail levels, loss of the endothelial marker PECAM1, an increase in the mesenchymal markers vimentin and α-SMA, and the morphological changes to a fibroblast phenotype. Together with the immunohistology data, our findings strongly suggest that Notch3 had driven EndMT which led the endothelial cells to acquire a mesenchymal cell phenotype and exhibit phenotypic properties of fibroblasts resulting to the eventual FO closure. Our data suggested a ∼70% transdifferentiation induced by Notch3 activation. Interestingly, we also observed a number of α-SMA positive cells that do not seem to have overt Notch3 staining in the N3ICD overexpressed cells. As we could not find any α-SMA positive cells in the non-N3ICD overexpressed control cell population, the appearance of α-SMA is likely related to Notch3 activation. Therefore, it is likely that the transdifferentiation efficiency is even higher than 70%, and Notch3 could induce endothelial cells to undergo EndMT at very low concentration, below the immunofluorescent detection threshold. To confirm these findings, a loss-of-function study is required to explore the exact role of Notch3 in this process. In addition, directly using endocardial cells rather than coronary artery endothelial cells, or cells derived from regional heart explant may be more informative.

We noticed that following FO closure, nuclear Snail at P7 could still be observed. Although Snail is a key transcription factor that controls EndMT, it also regulates cell proliferation, survival and migration [51], and is expressed in activated fibroblasts associating with increased proliferation and synthesis of ECM proteins [52]. Thus, it is possible that after FO closure, Snail may continue to play a role in atrial septal fibrosis by promoting ECM production.

In conclusion, we show that FO closure is a process of EndMT mediated fibrosis with the likely involvement of Notch signalling. Our data provide new perspectives on the physiological processes of postnatal atrial septal development. Our findings contribute to elucidating the molecular mechanisms of PFO development and may help to aid the future diagnosis and treatment of PFO-associated clinical conditions.
